# Internal rolling method for particle shape evaluation and reconstruction

**DOI:** 10.1371/journal.pone.0242162

**Published:** 2020-11-12

**Authors:** Pin-Qiang Mo

**Affiliations:** State Key Laboratory for GeoMechanics and Deep Underground Engineering, School of Mechanics and Civil Engineering, China University of Mining and Technology, Xuzhou, Jiangsu, China; Peking University, CHINA

## Abstract

A concise and robust method for 2D particle shape evaluation and reconstruction is proposed using the concept of the internal rolling of covering discs within the outline of a particle. By downscaling the covering disc size for capturing multiscale features, the calculation of the Euclidean distance can effectively yield three indices for sphericity, roundness and roughness. The geometric-based evaluations of particle morphology are dimensionless and precisely distinguishable between shapes after calibration and validation against constructed particles and natural sands. A sphericity-roundness diagram is provided to visualize the particle shape characterization, and a probabilistic density surface in the sphericity-roundness diagram is then proposed to statistically represent the distributions of the particle shapes. The concept of internal rolling is also utilized for particle shape reconstruction using a limited number of balls to replicate the indices of sphericity, roundness and roundness characteristic curve. The probabilistic density surface is duplicated for statistical particle shape reconstruction, which provides an effective approach for numerical investigations of the relationships between particle shapes and mechanical behavior. The effect of image quality on 2D shape evaluation is also examined by using a minimum area per particle, and the proposed method is intuitively extendable to 3D measurements using rolling covering spheres.

## Introduction

**Fig 1:**
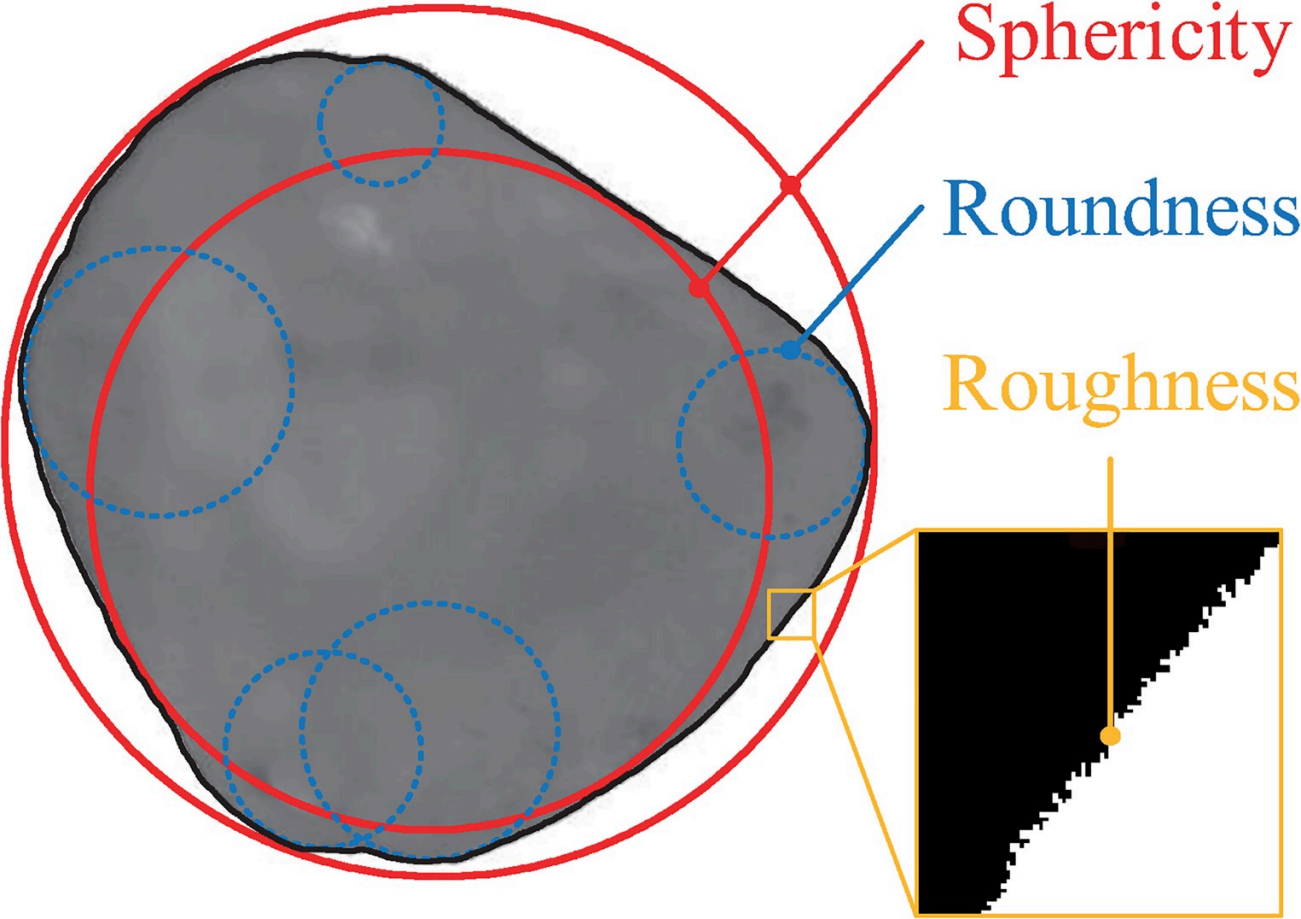
Schematic illustration of sphericity, roundness and roughness of a particle.

Particle shape is one of the most important inherent particle characteristics and is of interest to many fields of science and technology, from pharmacology and food engineering, to geoscience and geotechnical engineering. It has long been recognized that particle shape plays a major role in fabric formation (along with particle ordering and packing density) and thus influences the stress-strain behavior and strength properties of granular materials [1–3]. Definitions have been proposed for descriptive and quantitative representations of particle morphology, and it is well established that an irregular particle shape can be decomposed into three independent multiscale features: sphericity, roundness and roughness [4–10]. As depicted in Fig 1, sphericity describes the macroscale form of the particle compared to an inscribed or circumscribed sphere. Roundness refers to the mesoscale measurements of corners and edges obtained by quantifying the sharpness of angular protrusions for major surface features. Roughness delineates the microscale surface texture, capturing asperities that affect interparticle contact behavior.

Verbal descriptors and visual comparison charts have traditionally been used for manual measurements of particle shape with subjective biases and tedious efforts [5, 6]. Recent developments in imaging and computational techniques have facilitated the improvement of automated quantification methods for particle shape characterizations to remove the subjectivity inherent in the chart methods. For quantitative analyses, more complicated and sophisticated methods than their predecessors have also been employed to obtain accurate particle morphologies; these methods include scanning electron microscopy (SEM), photometric stereo acquisition [11], optical interferometry [12], microfocus X-ray computed-tomography [13, 14], and fuzzy uncertainty texture spectrum [15], along with the techniques of image segmentation, Fourier transformation, fractal analysis, Gaussian regression filtering, spherical harmonic analysis, and other hybrid algorithms. Nevertheless, there is a lack of a consistent approach for the quantification of particle shape, and this results in numerous vague and nonunified concepts. Specifically, corners are typically measured for describing roundness, whereas corner identification still requires manual criteria or mathematical functions with coefficients that lack physical meaning. The high scale-dependence of roughness results in empirical representations, and direct measurements of roughness are cumbersome [2]. Technically, Fourier analyses for unrolled particle outlines fail to furnish physical parameters for shape representation, and fractal dimensioning techniques have difficulties in distinguishing between particle shapes [16, 17]. Technologies for capturing three-dimensional particle shapes have been developed in recent decades to yield increasingly representative shape descriptors, especially for irregular granular particles. However, a 3D shape analysis typically requires sophisticated instruments and complex algorithms, and it is often difficult and inefficient to perform 3D characterization and particle reconstruction. Additionally, 2D shape descriptors have also been used to predict 3D geometrical features [1, 3, 14].

In this study, an attempt is made to propose a geometry-based method for particle shape evaluation without introducing complicated algorithms or coefficients. Sand particles are taken as a typical example in the subsequent analyses to demonstrate the proposed method, but the particle shape evaluation can be applied to an enormous variety of different granular materials with extensive implications. This paper focuses on 2D analysis, and the internal rolling method utilizes a covering disc to extensively occupy the area within the particle outline. As the size of the covering disc varies, the occupied areas are analyzed for descriptions of sphericity, roundness and roughness. This approach can be easily formulated by calculating the Euclidean distance, and it is critically analyzed for various irregular particles. The analyses are conducted only regarding these areas due to the intuitive information inherent in a rough surface and so that its fractured nature can be circumvented. The particle shape representation is concise and robust, and a statistical analysis is conducted to obtain the distributions of the descriptors. Additionally, a simple reconstruction method is also provided using the proposed indices and characteristic curve in an accurate and reliable manner.

### Internal rolling method for particle shape evaluation

**Fig 2:**
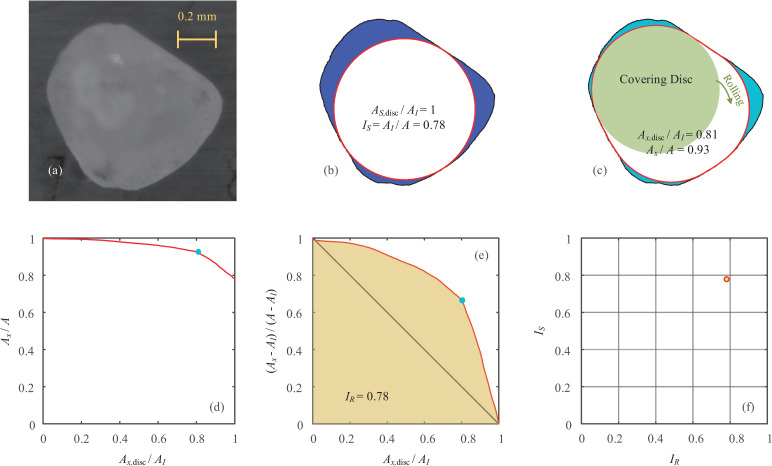
An example of particle shape evaluation: (a) microscope photograph of an Ottawa sand grain; (b) particle boundary, maximum inscribed circle and index of sphericity; (c) internal rolling by a given covering disc; (d) normalised covered area against normalised disc area; (e) roundness characteristic curve and index of roundness; (f) sphericity-roundness diagram.

To illustrate the particle shape evaluation, 2D microscope images of Ottawa sand are obtained for this study using a high-definition digital camera with a charge-coupled device (CCD) image sensor manufactured by SangNond, and a patch of one typical grain with a spatial resolution of approximately 1500 pixels/mm is selected as an example, as depicted in Fig 2A. Through digital image processing for a 2D projection of the particle profile, a binary particle silhouette is obtained for shape analysis. The particle outline is then extracted, and the maximum inscribed circle is determined and described using a Euclidean distance map (providing distance from each pixel to the closest outline), as shown in Fig 2B. Referring to the overall form of the particle by measuring the degree of conformity to a sphere/circle [12], the index of sphericity *I*_*S*_ is defined as:

$$I_{S}=\frac{A_{I}}{A}$$ (1)

where *A* is the particle area, and *A*_*I*_ is the area of the maximum inscribed circle. The value of *I*_*S*_ equals 1 for a circle and tends toward 0 for extremely irregular shapes. The index of sphericity avoids the use of particle perimeter, which is essentially more sensitive to image resolution. In this example, the *I*_*S*_ of the Ottawa sand grain is 0.78, compared to the sphericity with a range of 0.6–0.81 for Ottawa sand [16] and to the mean aspect ratio between 0.77 and 0.79 for Ottawa sand [18]. A validation against other descriptors in terms of sphericity is presented in a later section.

The measurement of roundness has been used to quantify the sharpness and curvature of the corners and edges since 1932 [4]. The widely used definition coined by Wadell [4] requires the evaluation of the average radius of curvature for all convex portions of the particle contour. However, the difficulties in corner identification lead to the measurement having reduced robustness, although methods using computational geometry were developed by introducing filtering techniques, inevitable corner thresholds and cutoff amplitudes [10, 17]. Therefore, a new method incorporating the internal rolling of covering disc is introduced in this study to intuitively describe the roundness of a particle.

A circular covering disc, which is not larger than the maximum inscribed circle, is placed and arbitrarily rolled within the particle outline. The maximum area covered by the internal rolling of the covering disc is recorded as *A*_*x*_. When the covering disc has the same area as the maximum inscribed circle (i.e., *A*_*x*,disc_ = *A*_*I*_), the covered area is always *A*_*x*_ = *A*_*I*_, and the evaluation of sphericity in Fig 2B is reproduced with an extreme covering disc. As the size of the covering disc is downscaled, the covered area increases with more rolling possibilities. Fig 2C shows a covering disc with *A*_*x*,disc_/*A*_*I*_ = 0.81, and the covered area ratio *A*_*x*_/*A* = 0.93 is higher than the value of *I*_*S*_.

Note that the area of the covering disc can vary across a range of 0<*A*_*x*,disc_≤*A*_*I*_, and the resulting covered area is in the range *A*>*A*_*x*_≥*A*_*I*_. The decrease in the ratio of the covered area to the normalized area of the covering disc is presented in Fig 2D, and the ultimate point approaches the index of sphericity. Note that the light blue point in the plot indicates the case with a specific covering disc, as shown in Fig 2C. To remove the influence of sphericity, the curve is then normalized in Fig 2E, where (*A*_*x*_−*A*_*I*_)/(*A*−*A*_*I*_) declines from 1 to 0. Taking the residual area produced by the maximum inscribed circle as a reference, the normalized covering area represents the ratio of the increased area to the residual area after the internal rolling of a given covering disc is completed. The geometric interpretation indicates that the increased area includes all corners with larger curvature values than those of the covering disc. Therefore, the normalized curve in Fig 2E is termed as the roundness characteristic curve (RCC), and the first integral is defined as the index of roundness:

$$I_{R}=\int_{0}^{1}\frac{A_{x}-A_{I}}{A-A_{I}}d\frac{A_{x,disc}}{A_{I}}$$ (2)

Similarly, *I*_*R*_ varies between 0 and 1, yielding lower values for more angular particles. The index of roundness for the Ottawa sand grain is 0.78, compared to the roundness values of 0.75 and 0.78 for Ottawa sand 20–30 [19, 20]. It is also worth noting that the internal rolling process can be realized efficiently and effectively through Euclidean distance mapping without introducing any subjective parameters.

The analyses of both sphericity and roundness are therefore coupled with the internal rolling method, and the magnitudes of *I*_*S*_ and *I*_*R*_ are essentially unique and independent; these two indices serve as reliable descriptors for quantitative shape evaluation. A sphericity-roundness (S-R) diagram is therefore provided in Fig 2F to investigate the distributions of *I*_*S*_ and *I*_*R*_. The regularity of the particle shape decreases nonlinearly with the distance to the right-top point for a sphere/circle in the S-R diagram.

### Calibration and validation

**Fig 3:**
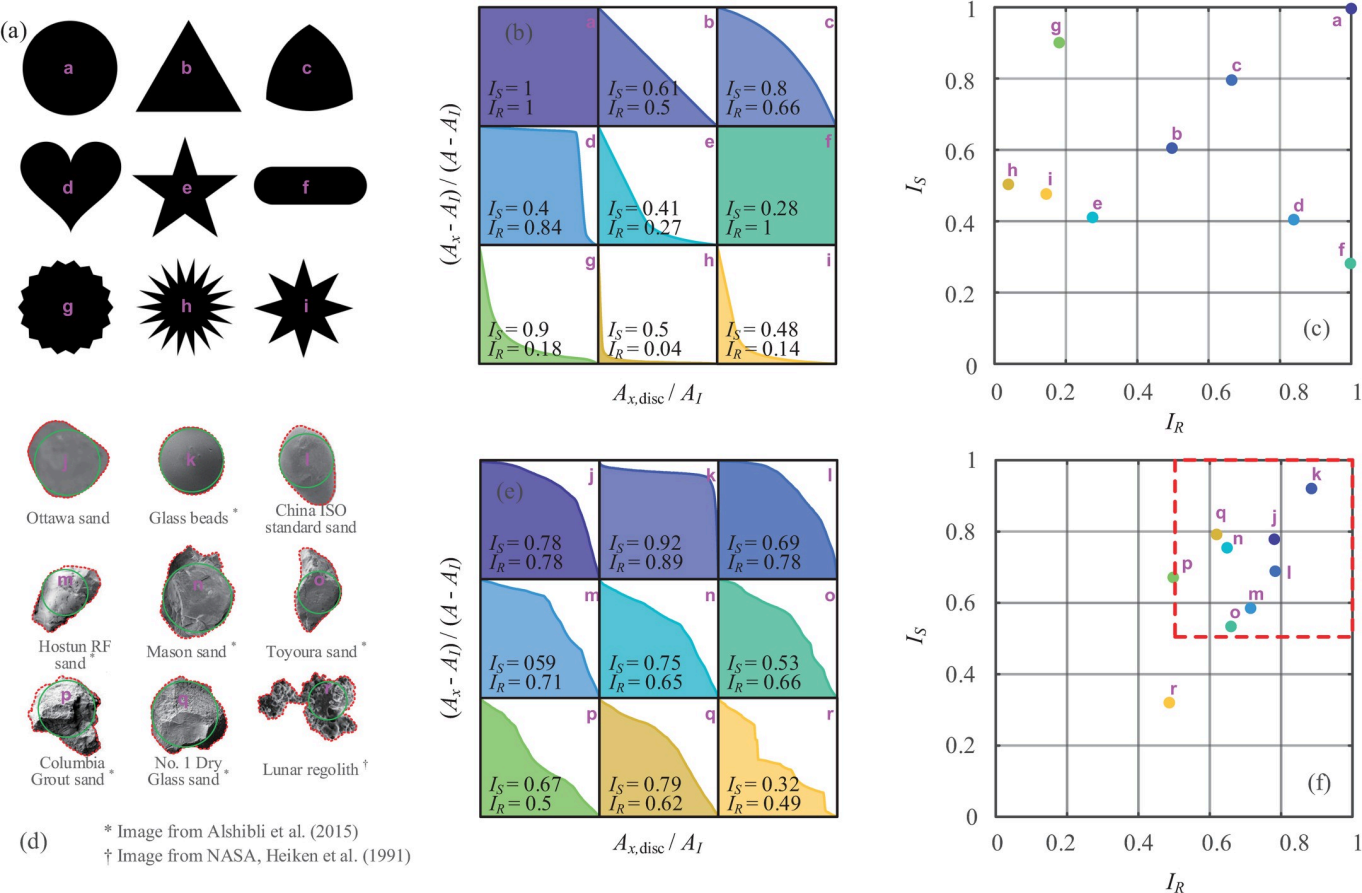
Calibration of particle shape evaluation: (a) constructed geometries: Particles a~i; (b) RCCs for Particles a~i; (c) S-R diagram for Particles a~i; (d) typical sands: Particles j~r; (e) RCCs for Particles j~r; (f) S-R diagram for Particles j~r.

To calibrate the proposed particle shape evaluation method, nine idealized geometric particles are purposely constructed in Fig 3A with various shapes, verifying the high robustness of the internal rolling method. The roundness characteristic curves (RCCs) with corresponding values of *I*_*S*_ and *I*_*R*_ are collectively provided in Fig 3B, and the sphericity-roundness (S-R) diagram is plotted in Fig 3C. The circle (Particle a: *I*_*S*_ = 1 and *I*_*R*_ = 1) serves as the extrema of both sphericity and roundness. The *I*_*S*_ of the regular triangle (Particle b) is very close to the theoretical value of $\sqrt{3}\pi /9$, and its *I*_*R*_ equals 0.5, corresponding to the isosceles-right-triangle shaped RCC. Theoretically, an *I*_*R*_ =0.5 and an isosceles-right-triangle shaped RCC are features of all regular polygons, so these can be treated as a reference (also seen in Fig 2E). The Reuleaux triangle (Particle c) gives higher *I*_*S*_ and *I*_*R*_ values than those of Particle b, and this is attributed to the curved edges. Particles d and e have comparable *I*_*S*_ values, whereas their *I*_*R*_ values are very different, and the pentagram is apparently more angular. It is interesting to see that the *I*_*R*_ values of capsule shapes (e.g. Particle f) remain at 1 since the shrunken maximum inscribed circle could cover all of the particle area, and *I*_*S*_ becomes the main descriptor for the form of the particle. On the other hand, Particles g-i are created with *I*_*R*_<0.2, and the spikes represent the corners of irregular particles. *I*_*S*_ generally decreases with the spike length, and *I*_*R*_ seems to decrease with the number of spikes. The constructed ideal shapes are distributed widely in the S-R diagram, and the proposed RCC and S-R diagram can precisely describe the sphericity and roundness of essentially any 2D solid particle.

In terms of the soil mass, individual grains of 7 typical sands, glass beads [21] and lunar regolith [22] are randomly selected for comparison purposes (Fig 3D–3F). The glass bead (Particle k) exhibit a very circular shape with *I*_*S*_ = 0.92 and *I*_*R*_ = 0.89, and most natural sands are located in the zone of *I*_*S*_>0.5 and *I*_*R*_>0.5 in the S-R diagram. The Columbia grout sand (Particle p) is the most angular sand, and the Toyoura sand (Particle o) is the most eccentric among the typical sands. By contrast, the lunar regolith (Particle r) is an irregular agglutinate from soil 10084, and its indices (*I*_*S*_ = 0.32 and *I*_*R*_ = 0.49) appear to bear out the irregular shape with sharp corners [22]. The calibration tends to indicate that natural sands formed by weathering are mostly granular with *I*_*S*_>0.5 and *I*_*R*_>0.5, especially under conditions of erosion and transportation due to waves and wind. However, considering the high irregularity of particle shapes, the full S-R diagram is useful for representation.

**Fig 4:**
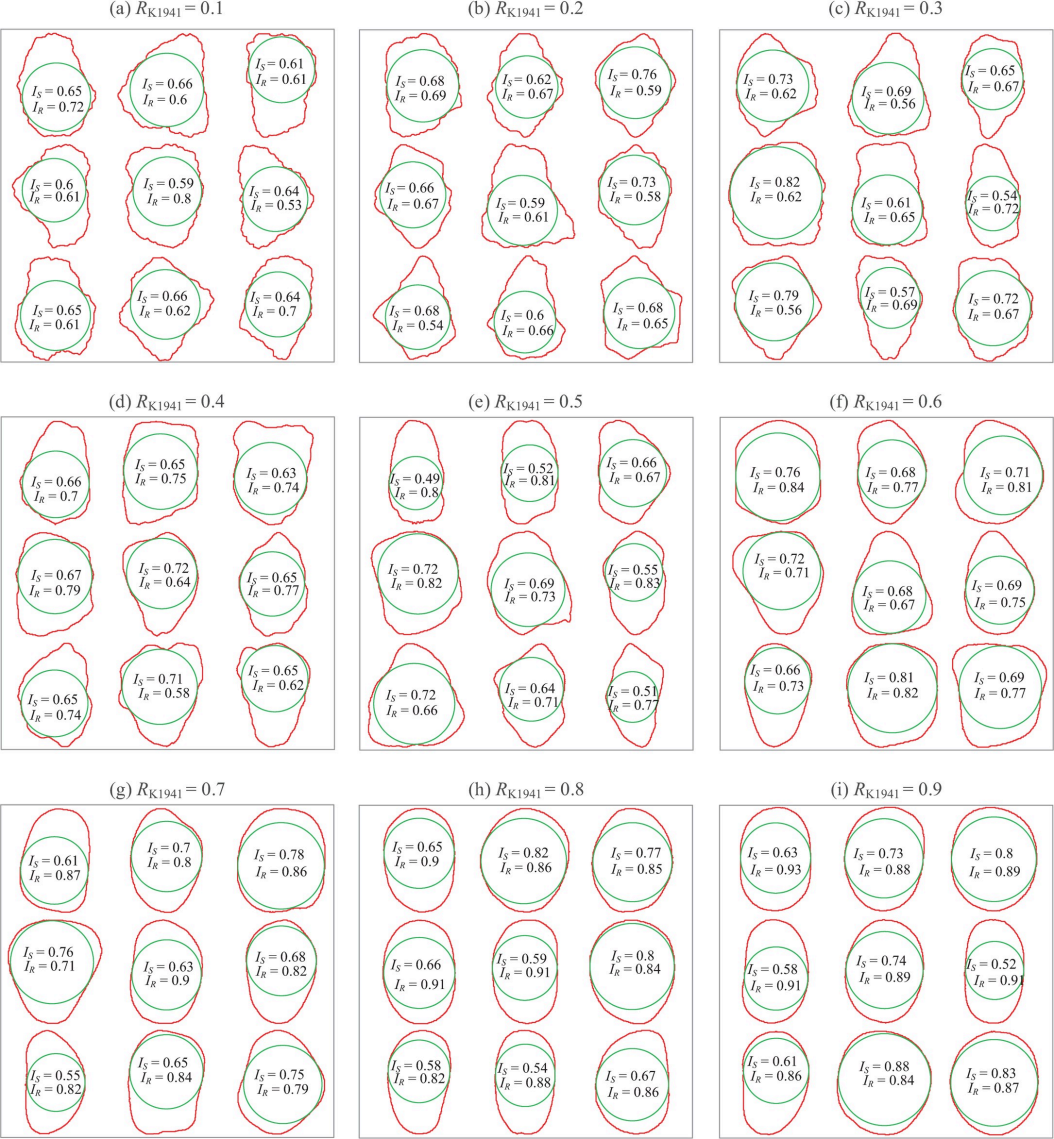
Indices of sphericity and roundness for particles from Krumbein [5].

For validation against existing particle shape evaluation techniques, the proposed method is implemented on the comparison roundness chart for pebbles [5]. The 81 particles are classified into 9 bins with different roundness values (*R*_K1941_) ranging from 0.1 to 0.9, and these bins were manually evaluated by Wadell’s definition [4]. The boundaries of all particles are redrawn with their maximum inscribed circles, and the computed indices of *I*_*S*_ and *I*_*R*_ are also presented for the particles in Fig 4.

**Fig 5:**
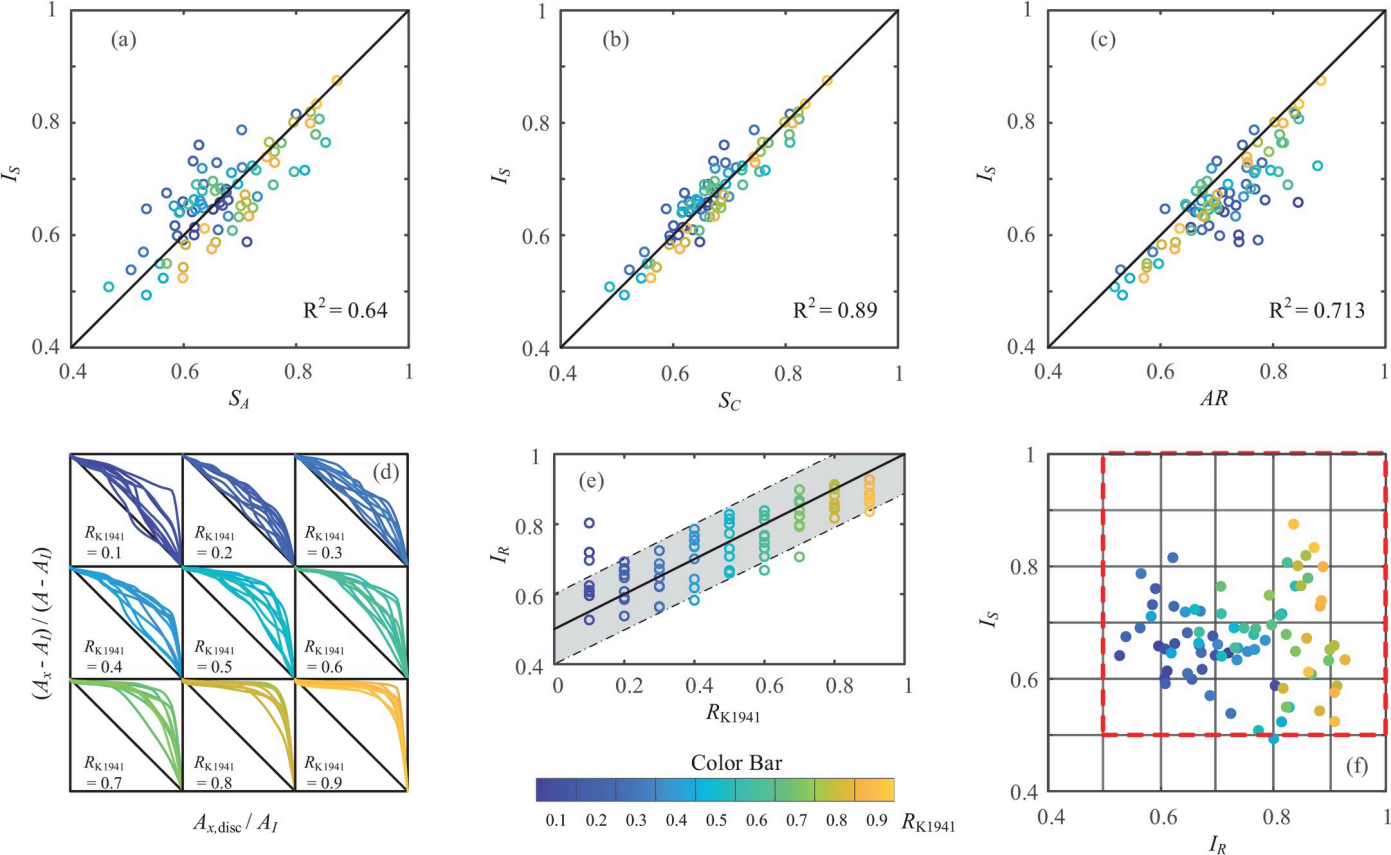
Validation of particle shape evaluation: (a) comparisons between *I*_*S*_ and *S*_*A*_; (b) comparisons between *I*_*S*_ and *S*_*C*_; (c) comparisons between *I*_*S*_ and *AR*; (d) RCCs; (e) comparisons between *I*_*R*_ and *R*_K1941_; (f) particles in S-R diagram.

In terms of the index of sphericity, the values of *I*_*S*_ for all 81 particles are compared those obtained with three other commonly used descriptors of sphericity, namely, the area sphericity [23], circle ratio sphericity [1] and aspect ratio [24], and their definitions are listed respectively:

$$S_{A}=\frac{A}{A_{E}}$$ (3A)

$$S_{C}=\sqrt{\frac{A_{I}}{A_{E}}}$$ (3B)

$$AR=\frac{D_{F,min}}{D_{F,max}}$$ (3C)

where *A*_*E*_ is the area of the minimum circumscribed circle; *D*_*F*,min_ and *D*_*F*,max_ are the minimum and maximum Feret diameters, respectively. The Feret diameter or caliper diameter measures the perpendicular distance between parallel tangents touching opposite sides of the particle outline [25]. Note that *S*_*A*_ denotes circularity [26]; *I*_*S*_ is thus identical to *S*_*C*_^2^/*S*_*A*_; and the definition of *AR* is similar but slightly different from the elongation [26], width to length ratio sphericity [7], and aspect ratio [27]. The comparisons between the sphericities are shown in Fig 5A–5C. It is obvious that the results of *I*_*S*_ are generally in good agreement with all three descriptors of sphericity, especially for *S*_*C*_.

Fig 5D collectively plots all RCCs in the nine bins for evaluating *I*_*R*_. The RCCs have similar trends for particles from the same bin in Fig 4, and the profile tends to deviate from the diagonal line and approach the extrema based on the circles’ coordinates as the value of *R*_K1941_ increases from 0.1 to 0.9. The profile of the RCC above the diagonal line again indicates the reference value of *I*_*R*,ref_ = 0.5 from regular polygons. The comparisons of *I*_*R*_ and *R*_K1941_ are provided in Fig 5E, showing a notable positive correlation. A simple relationship can thus be used to correlate the *I*_*R*_ and *R* values based on Wadell’s concept, and this relationship is given as *I*_*R*_ = *R*/2+0.5 (depicted in Fig 5E). The minimum value of *I*_*R*_ from Krumbein’s chart verifies the narrowed zone in the S-R diagram for natural sands, as shown by the red frame in Fig 5F. It is noteworthy that the pebble shapes in Krumbein’s chart are mainly convex. Defining the convexity (*C*) as the ratio of the particle area to the convex hull of the particle, the magnitude of *C* falls in 0.92 ~ 0.99 for all particles in Fig 4. It is predictable that lower values of *I*_*S*_ and *I*_*R*_ are attainable outside of the narrowed zone for particles with less convexity, as can be seen in Fig 3 for Particles e, h, i and r. Therefore, the definition of *I*_*R*_ in this study extends the distribution of roundness based on Wadell’s concept, and the proposed S-R diagram is useful for highly general and broadly applicable particle shape characterizations.

### Statistical evaluation of sphericity and roundness

**Fig 6:**
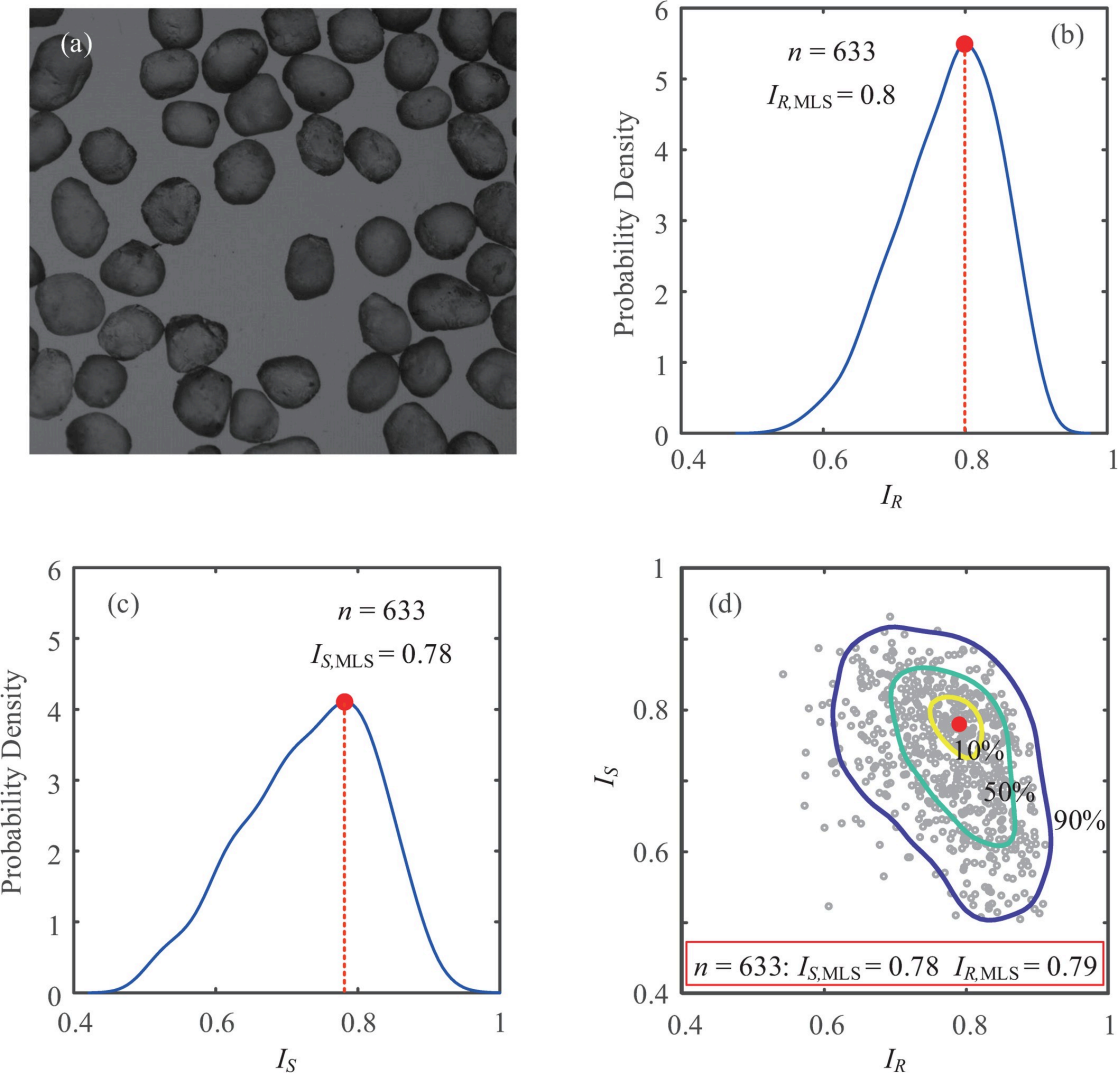
Statistical evaluation of particle shape: (a) a patch of microscope photograph for Ottawa sand; (b) PDC of *I*_*R*_; (c) PDC of *I*_*S*_; (d) PDS in S-R diagram.

The intrinsic natural variability of geomaterials results in variations in *I*_*S*_ and *I*_*R*_ from particle to particle. A statistical evaluation for a type of granular matter is essentially necessary to obtain the valid distributions of shape descriptors. To achieve a statistically representative evaluation of particle shape, populations of greater than 30~50 particles are required [28, 29]. In this study, a total of 633 particles are randomly recognized from microscope images of Ottawa sand for statistical analysis, and a patch of particle assemblies is shown in Fig 6A.

After the fast process of the proposed shape evaluation, a kernel smoothing technique is applied to obtain a continuous statistical variable, and the probabilistic density curves (PDC) for both *I*_*R*_ and *I*_*S*_ are then presented in Fig 6B and 6C. The total area under the density curve is 1, and the PDCs appear to be follow nonnormal distributions. The peak of each PDC represents the maximum likelihood score (MLS) of the distribution, and *I*_*S*,MLS_ = 0.78 and *I*_*R*,MLS_ = 0.8 are obtained from the PDCs.

Despite the independent definitions of *I*_*S*_ and *I*_*R*_, the properties of particle assemblies are not statistically independent, and a probabilistic density surface (PDS) is more reasonable than PDCs to reveal the distributions in the sphericity-roundness (S-R) diagram (Fig 6D). From the PDS, the MLS values are *I*_*S*,MLS_ = 0.78 and *I*_*R*,MLS_ = 0.79. Contours with certain probabilities are also shown in Fig 6D, indicating that 10%, 50% and 90% of particles fall within these zones. It needs to be emphasized that the PDS in the S-R diagram is more statistically representative of particle shape evaluation than of unique values of sphericity and roundness.

### Particle shape reconstruction

Particle shape representation and reconstruction are becoming increasingly important for numerical investigations of particle morphology and its effects on micro-macro mechanical behavior [1, 30–32]. Clumps, clusters and polyhedrons have typically been used in discrete element modeling to represent a realistic shape, along with developed overlapping or skeletonization algorithms (e.g. [26, 33–35]). Particle reconstruction using clumps is arguably the most computationally efficient method owing to its domain overlapping filling, while the approach of replicating a single particle with over 100 balls (spheres or discs) is currently infeasible due to its excessive computational cost [36].

**Fig 7:**
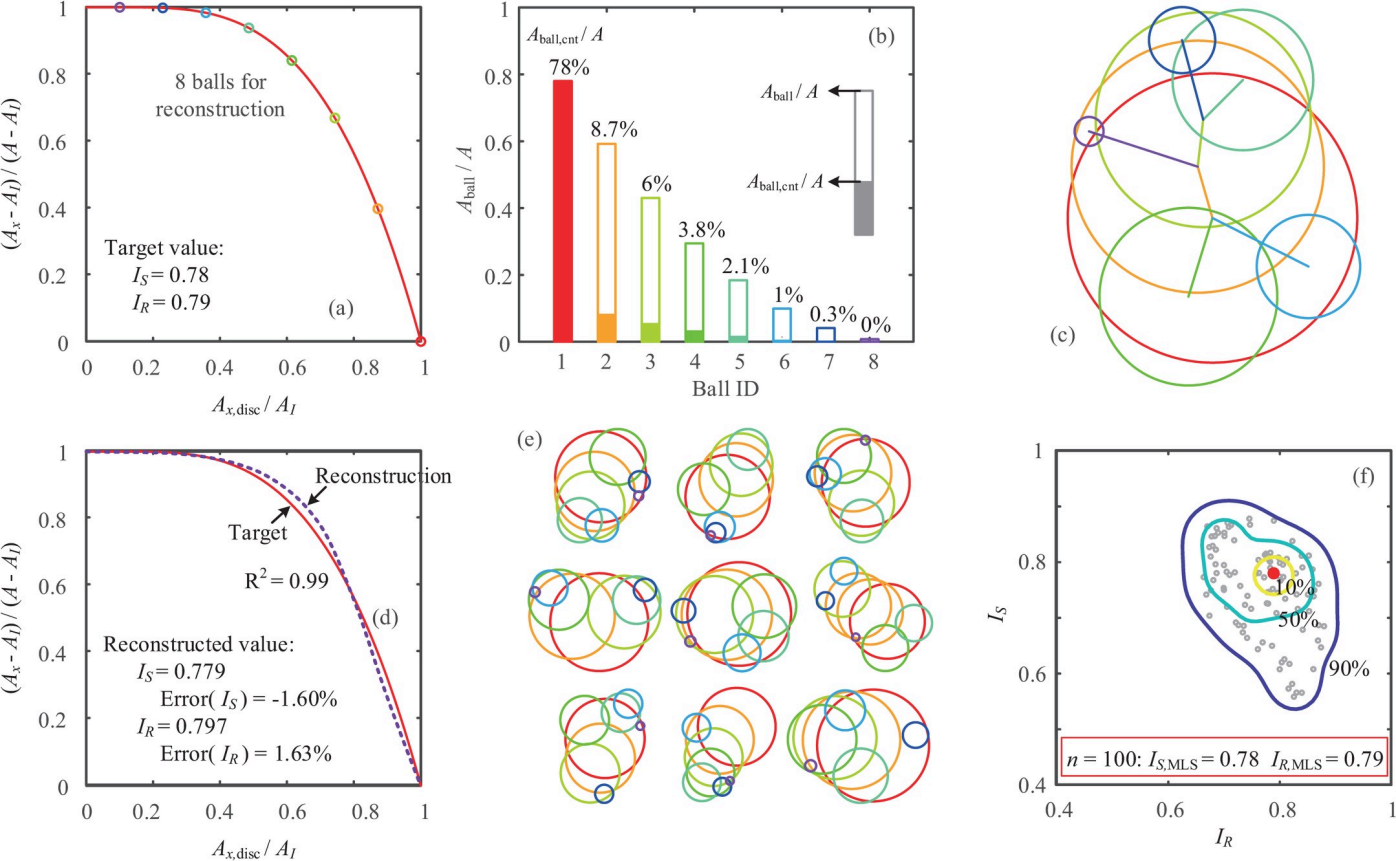
Particle shape reconstruction for Ottawa sand: (a) target value and estimated RCC with 8 balls; (b) ball areas and their contributions; (c) reconstructed particle with random ball positions; (d) reconstructed particle shape indices and RCC; (e) particle shape reconstruction with a given PDS from Fig 6D; (f) PDS for 100 reconstructed particles.

In this study, a particle shape reconstruction method is proposed for representing particles with identical indices of sphericity and roundness and roundness characteristic curve (RCC), using a limited number of balls. For a given pair of indices with *I*_*S*_ = 0.78 and *I*_*R*_ = 0.79 (derived from the maximum likelihood score (MLS) of Ottawa sand), the RCC is first produced from either experimental measurements or mathematical estimations (e.g. $y=1-x^{I_{R}/(1-I_{R})}$), as shown in Fig 7A. A small number of balls is assigned to fulfil the particle representation, and an example with *n*_ball_ = 8 is illustrated in Fig 7. The RCC is used to distribute the ball sizes with *A*_ball_ and to determine the areas distributing to the particle (*A*_ball,cnt_). Fig 7B lists the balls in order of their sizes and area contributions. The smaller balls are then successively added to a random possible ball in the clump at a random available position. The balls’ center distance is determined by the radii of two balls and the contributing area of the smaller ball. Eventually, the particle with eight balls is reconstructed randomly, and its *I*_*S*_, *I*_*R*_ and RCC are replicated, as depicted in Fig 7C. A validation is then conducted to evaluate the reconstructed particle shape using the internal rolling method. The comparable RCCs are shown in Fig 7D with *R*^2^ = 0.99, and the indices of both *I*_*S*_ and *I*_*R*_ are perfectly reproduced with less than a 2% error rate. Generally, 5~10 balls are recommended to obtain a good representation of particle shape, whereas with a smaller number of balls, it is still possible to satisfy *I*_*S*_ with less accuracy in terms of the *I*_*R*_ and RCC.

Additionally, the RCC reconstruction method can also incorporate the PDS of the descriptors to statistically replicate particle shapes in an efficient and extensible process. When the inputs of *I*_*S*_ and *I*_*R*_ are statistically designated according to the PDS in the S-R diagram from Fig 6D for Ottawa sand, the assembly of clumps is automatically generated, representing realistic granules. Nine examples are randomly selected and delineated in Fig 7E, and 100 particles are rapidly reconstructed with an almost identical PDS compared to that measured from the microscope images (Fig 7F). The RCC reconstruction turns out to be an effective tool for generating numerous particles with realistic shape distributions in a precise and robust manner for numerical simulations of granular materials. It should be noted that the proposed reconstruction method cannot replicate all morphometric features of particles, and reconstructed particles with identical indices of sphericity and roundness might exhibit different behavior than those of real particles. Further investigation with a corresponding numerical study is required to validate the applicability of the proposed method for modeling the mechanical features of granular materials.

## Discussion

### Effects of image quality on shape evaluation

For image-based analyses of particle shapes, image quality is a critical factor that affects the evaluation. Numerous indicators have been proposed to quantify the image resolution of a particle including the pixels per minimum Feret dimeter [18], circumscribed circle diameter [10], particle lengths and perimeter [37]. This study utilizes the area-based analysis owing to its uniqueness and less sensitivity (compared to the scale-dependent perimeter and the direction-dependent diameter), and the area per particle (APP) is suggested to represent the image quality for particle shape analysis. By comparing existing studies, it can be seen that the square root of the APP is somehow equivalent to the particle length.

**Fig 8:**
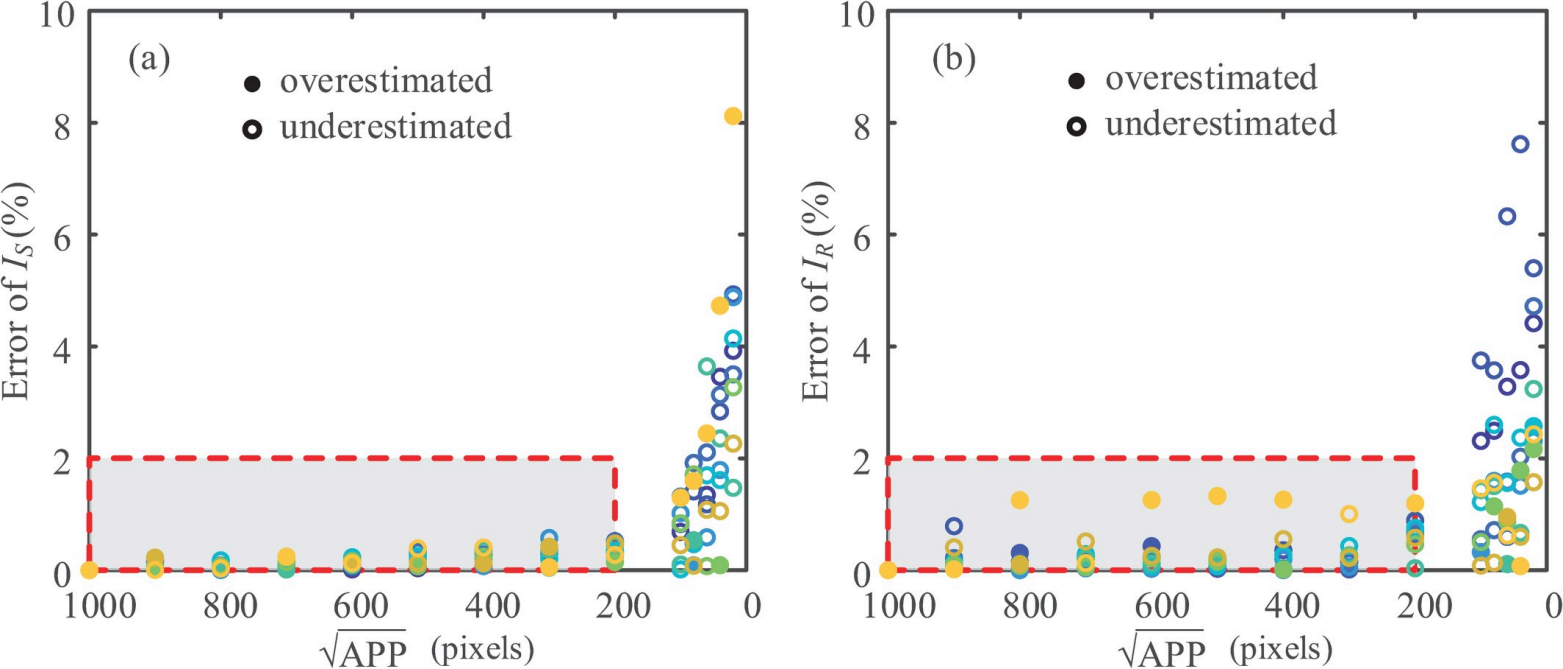
Effects of $\sqrt{APP}$ on particle shape indices: (a) error of *I*_*S*_; (b) error of *I*_*R*_.

The effects of $\sqrt{APP}$ on the particle shape indices *I*_*S*_ and *I*_*R*_ are shown in Fig 8 for the 9 sand particles from Fig 3D. Acknowledging the acceptable 2% error rate, the minimum image quality yields ${\sqrt{APP}}_{min}\approx 200\;pixels$, which agrees well with the previous criteria [10, 37].

### Evaluation of roughness

**Fig 9:**
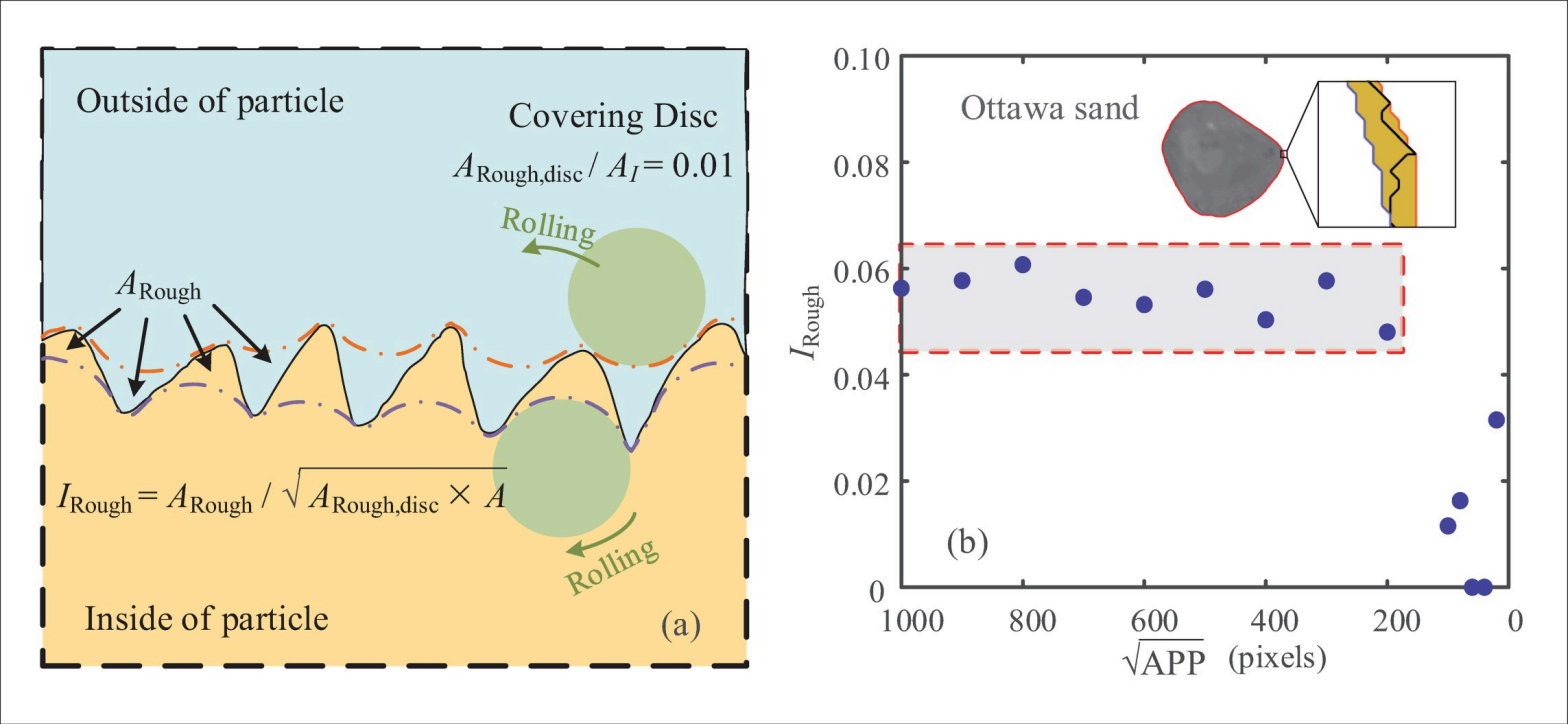
Evaluation of roughness: (a) illustration of covering discs inside and outside of particle boundary; (b) *I*_Rough_ of an Ottawa sand grain against $\sqrt{APP}$.

Roughness describes the microscale features of the particle surface. Measurements from conventional image photogrammetry, however, are fairly cumbersome [2], and values of roughness are scale dependent and highly sensitive to image resolutions. Filtering techniques have typically been adopted to remove roughness features and obtain a smoothed particle boundary, which is then used for the evaluation of sphericity and roundness. To extend the aforementioned approach, the proposed rolling method for shape evaluation is also applicable to the description of roughness by both internal and external rolling of a small covering disc that can capture features of asperities for roughness. Therefore, the schematic of roughness evaluation is illustrated in Fig 9A, and the index of roughness *I*_Rough_ is defined as:

$$I_{Rough}=\frac{A_{Rough}}{\sqrt{A_{Rough,disc}\times A}}\leq 1$$ (4)

where *A*_Rough,disc_ is the area of the roughness covering disc, which is 1% area of the maximum inscribe circle; and *A*_Rough_ is the area between boundaries formed by both internal and external rolling of the roughness covering disc. The variation in *I*_Rough_ relative to the image quality $\sqrt{APP}$ for the Ottawa sand particle in Fig 9B shows that the value of *I*_Rough_ is still stable for $\sqrt{APP}>200\;pixels$, despite the relatively high amount of errors. This method for evaluating roughness seems to be intuitive with explicit physical meaning. However, the limitation caused by the image quality constraint is questionable, and further study is required to segment the noise of digital images from the roughness features.

### Extension to 3D shape evaluation and reconstruction

This study focuses on 2D particle shape evaluation using the proposed internal rolling method for quantitative characterizations of sphericity, roundness and roughness, as well as the 2D particle reconstruction method utilizing the RCC and PDS in the S-R diagram. Although the 2D method remains the most effective and accurate approach, 3D morphological analysis is increasingly performed to fully represent particle characteristics [2, 14, 38–40]. For this reason, the proposed method in this study is essentially generic and adaptable; it can be easily extended to 3D analyses for both evaluation and reconstruction using the volumetric information and the concept of internal rolling spheres (Eq 5). This extension will soon be conducted in further studies.

$$I_{S,3D}=\frac{V_{I}}{V}$$ (5A)

$$I_{R,3D}=\int_{0}^{1}\frac{V_{x}-V_{I}}{V-V_{I}}d\frac{V_{x,sphere}}{V_{I}}$$ (5B)

$$I_{Rough,3D}=\frac{V_{Rough}}{\sqrt{V_{Rough,sphere}\times V}}\leq 1$$ (5C)

where *I*_*S*,3D_ is the index of sphericity for 3D particles, *V* is the particle volume, and *V*_*I*_ is the volume of the maximum inscribed sphere; *I*_*R*,3D_ is the index of roundness for 3D particles, *V*_*x*_ is the maximum covered volume by internal rolling of the covering sphere with a volume of *V*_*x*,sphere_; *I*_Rough,3D_ is the index of roughness for 3D particles, *V*_Rough,sphere_ is the volume of the roughness covering sphere, which is 1% volume of the maximum inscribe sphere; and *V*_Rough_ is the volume between boundaries formed by both internal and external rolling of the roughness covering sphere.

## Summary and conclusions

An internal rolling method for particle shape evaluation is proposed in this study using various sizes of covering discs within the 2D particle outline. The concept of internal rolling is intuitive, rigorous, and valid for capturing all three multiscale features, as summarized below:

1. Sphericity. When the covering disc is identical to the maximum inscribed circle, the ratio of its occupied area to the particle area is defined as the index of sphericity, which measures the degree of conformity to a sphere.
2. Roundness. When downscaling the covering disc from the maximum inscribed circle to 1% of its area, the covered area increases with the decrease in the disc area, and the normalized relation is obtained as a unique roundness characteristic curve. The integration of the roundness characteristic curve (RCC), obtained by evaluating the corners at different scales, is thus defined as the index of roundness.
3. Roughness. The smallest covering disc with 1% of *A*_*I*_ is again used for both internal and external rolling along the particle boundary, and the trimmed area representing the asperities is then taken for the evaluation of roughness.

The descriptors yielded from the rolling method are both concise and independent, furnishing geometric-based evaluations of particle morphologies. They are dimensionless, and the normalized values are capable of precisely distinguishing between shapes. Nevertheless, the measurements are also concise and robust, and they do not introduce complicated algorithms or empirically-based coefficients.

After the implementation of the internal rolling method for shape evaluation is completed, a sphericity-roundness diagram is provided to visualize the particle shape characterization, and a probabilistic density surface in the sphericity-roundness (S-R) diagram is then suggested to statistically represent the distributions of the particle shapes.

Particle shape reconstruction is effectively realized by utilizing the concept of internal rolling and the proposed PDS in the S-R diagram. A limited number of balls are required to replicate the indices of sphericity, roundness and RCC well. The indices-based reconstruction method will promote the investigation of the relationships between particle shape and mechanical behavior using discrete element simulation methods.

By examining the effects of image quality on 2D shape evaluation, it is found that the square root of the area per particle should be larger than 200 pixels to guarantee the precision of the descriptors for particle shape. The proposed method is also extendable to 3D measurements due to its good interpretation of basic physical information for quantitative shape analyses.

## References

[1] Santamarina J. C. & Cho G. C. (2004). Soil behaviour: The role of particle shape Advances in Geotechnical Engineering: The Skempton Conf., Thomas Telford, London, 604–617.

[2] Cho G. C., Dodds J. & Santamarina C. (2006). Particle shape effects on packing density, stiffness, and strength: natural and crushed sands. *J*. *Geotech*. *Geoenviron*. *Eng*. 132, No. 5, 591–602 10.1061/(ASCE)1090-0241(2006)132:5(591).

[3] Alshibli K. A. & Cil M. B. (2018). Influence of particle morphology on the friction and dilatancy of sand. *J*. *Geotech*. *Geoenviron*. *Eng*. 144, No. 3, 04017118 10.1061/(ASCE)GT.1943-5606.0001841

[4] Wadell H. (1932). Volume, shape, and roundness of rock particles. *J*. *Geol*. 40, No. 5, 443–451. 10.1086/623964

[5] Krumbein W. C. (1941). Measurement and geological significance of shape and roundness of sedimentary particles. *J*. *Sediment*. *Petrol*. 11, No. 2, 64–72. 10.1306/D42690F3-2B26-11D7-8648000102C1865D

[6] Powers M. C. (1953). A new roundness scale for sedimentary particles. *J*. *Sediment*. *Petrol*. 23, No. 2, 117–119. 10.1306/D4269567-2B26-11D7-8648000102C1865D

[7] Krumbein W. C. & Sloss L. L. (1963). Stratigraphy and sedimentation, 2nd Ed., Freeman and Company, San Francisco.

[8] Barrett P. J. (1980). The shape of rock particles, a critical review. *Sedimentology* 27, 291–303. 10.1111/j.1365-3091.1980.tb01179.x

[9] Blott S. J. & Pye K. (2008). Particle shape: a review and new methods of characterization and classification. *Sedimentology* 55, 31–63. 10.1111/j.1365-3091.2007.00892.x

[10] Zheng J. & Hryciw R. D. (2015). Traditional soil particle sphericity, roundness and surface roughness by computational geometry. *Géotechnique* 65, No. 6, 494–506. 10.1680/geot.14.P.192

[11] Smith M. L. (1999). The analysis of surface texture using photometric stereo acquisition and gradient space domain mapping. *Image Vis*. *Comput*. 17, No. 14, 1009–1019. 10.1016/S0262-8856(99)00003-7

[12] Alshibli K. A. & Alsaleh M. (2004). Characterizing surface roughness and shape of sands using digital microscopy. *J*. *Comput*. *Civ*. *Eng*. 18, No. 1, 36–45 10.1061/(ASCE)0887-3801(2004)18:1(36)

[13] Stock S. R. (2008). Recent advances in X-ray microtomography applied to materials. *Int*. *Mater*. *Rev*. 53, No. 3, 129–181. 10.1179/174328008X277803

[14] Su D. & Yan W. M. (2019). Prediction of 3D size and shape descriptors of irregular granular particles from projected 2D images. Acta Geotechnica. 10.1007/s11440-018-0749-z 32685053 PMC7357729

[15] Lee Y. G., Lee J. H. & Hsueh Y. C. (1998). Texture classification using fuzzy uncertainty texture spectrum. *Neurocomputing* 20, 115–122. 10.1016/S0925-2312(97)00095-7

[16] Sukumaran B. & Ashmawy A. K. (2001). Quantitative characterization of the geometry of discrete particles. *Géotechnique* 51, No. 7, 619–627. 10.1680/geot.2001.51.7.619

[17] Vangla P., Roy N. & Gali M. L. (2018). Image based shape characterization of granular materials and its effect on kinematics of particle motion. Granular Matter 20, No. 6 10.1007/s10035-017-0776-8

[18] Altuhafi F., O’Sullivan C. & Cavarretta I. (2013). Analysis of an image-based method to quantify the size and shape of sand particles. *J*. *Geotech*. *Geoenviron*. *Eng*. 139, No. 8, 1290–1307. 10.1061/(ASCE)GT.1943-5606.0000855

[19] Hryciw R. D., Zheng J. & Shetler K. (2016). Particle roundness and sphericity from images of assemblies by chart estimates and computer methods. *J*. *Geotech*. *Geoenviron*. *Eng*. 142, No. 9, 04016038 10.1061/(ASCE)GT.1943-5606.0001485

[20] DeJong J. T. & Christoph G. G. (2009). Influence of particle properties and initial specimen state on one-dimensional compression and hydraulic conductivity. *J*. *Geotech*. *Geoenviron*. *Eng*. 135, No. 3, 449–454. 10.1061/(ASCE)1090-0241(2009)135:3(449).

[21] Alshibli K. A., Druckrey A. M., Al-Raoush R. I., Weiskittel T. & Lavrik N. V. (2015). Quantifying Morphology of Sands Using 3D Imaging. *J*. *Mater*. *Civ*. *Eng*. 27, No. 10: 04014275 10.1061/(ASCE)MT.1943-5533.0001246

[22] Heiken G. H., Vaniman D. T. & French B. M. (1991). Lunar Sourcebook: A User’s Guide to the Moon. Cambridge University Press, London.

[23] Riley N. A. (1941). Projection sphericity. *J*. *Sediment*. *Petrol*. 11, No. 2, 94–95. 10.1306/D426910C-2B26-11D7-8648000102C1865D

[24] Cavarretta, I. (2009). The influence of particle characteristics on the engineering behaviour of granular materials. PhD thesis, Dept. Civil and Environmental Engineering, Imperial College London, London.

[25] Walton W. H. (1948). Feret’s Statistical Diameter as a Measure of Particle Size. *Nature* 162, 329–330.

[26] Mollon G. & Zhao J. D. (2012). Fourier-Voronoi-based generation of realistic samples for discrete modelling of granular materials. *Granular Matter* 14, No. 5, 621–638. 10.1007/s10035-012-0356-x

[27] Rickman D., Lohn-Wiley B., Knicely J. & Hannan B. (2016). Probabilistic solid form determined from 2D shape measurement. *Powder Technology* 291, 466–472. 10.1016/j.powtec.2015.10.044

[28] Bareither C. A., Edil T. B., Benson C. H. & Mickelson D. M. (2008). Geological and physical factors affecting the friction angle of compacted sands. *J*. *Geotech*. *Geoenviron*. *Eng*. 134, No. 10: 1476–1489. 10.1061/(ASCE)1090-0241(2008)134:10(1476)

[29] Zheng J. & Hryciw R. D. (2016). Roundness and sphericity of soil particles in assemblies by computational geometry. *J*. *Comput*. *Civ*. *Eng*. 20, No. 6 10.1061/(ASCE)CP.1943-5487.0000578

[30] Lai Z. & Chen Q. (2017). Characterization and discrete element simulation of grading and shape-dependent behavior of JSC-1A Martian regolith simulant. *Granular Matter* 19, 69 10.1007/s10035-017-0754-1

[31] Gao Y., Zhao C., Markine V., Jing G. & Zhai W. (2020). Calibration for discrete element modelling of railway ballast: A review. Transportation Geotechnics 23, 100341 10.1016/j.trgeo.2020.100341

[32] Gong J., Nie Z., Zhu Y., Liang Z. & Wang X. (2019). Exploring the effects of particle shape and content of fines on the shear behavior of sand-fines mixtures via the DEM. *Computers and Geotechnics* 106, 161–176.

[33] Das, N. (2007). Modeling three-dimensional shape of sand grains using discrete element method. PhD thesis, University of South Florida.

[34] Shi C., Li D., Xu W. & Wang R. (2015). Discrete element cluster modeling of complex mesoscopic particles for use with the particle flow code method. *Granular Matter* 17, No. 3, 377–387. 10.1007/s10035-015-0557-1

[35] Kawamoto R., Andò E., Viggiani G. & Andrade J. E. (2016). Level set discrete element method for three-dimensional computations with triaxial case study. *J*. *Mech*. *Phys*. *Solids* 91, 1–13. 10.1016/j.jmps.2016.02.021

[36] Ferellec J. & McDowell G. (2010). Modelling realistic shape and particle inertia in DEM. *Géotechnique* 60, No. 3, 227–32. 10.1007/s10035-010-0205-8

[37] Sun Q., Zheng J., Coop M. R. & Altuhafi F. N. (2019). Minimum image quality for reliable optical characterizations of soil particle shapes. *Computers and Geotechnics* 114: 103110 10.1016/j.compgeo.2019.103110

[38] Katagiri J., Matsushima T., Yamada Y., Tsuchiyama A., Nakano T., Uesugi K., et al (2015). Investigation of 3D Grain Shape Characteristics of Lunar Soil Retrieved in Apollo 16 Using Image-Based Discrete-Element Modeling. *J*. *Aerosp*. *Eng*. 28, No. 4, 04014092 10.1061/(ASCE)AS.1943-5525.0000421

[39] Zhou B. & Wang J. (2017). Generation of a realistic 3D sand assembly using X-ray microcomputed tomography and spherical harmonic-based principal component analysis. Int. J. Numer. Anal. Meth. Geomech. 41, 93–109. 10.1002/nag.2548

[40] Wang X., Su D. & Zhao J. D. (2019). Superellipsoid-based study on predicting 3D particle geometry from 2D projections. *Computers and Geotechnics* 114, 103131.

